# Genetic dissection of MHC-associated susceptibility to *Lepeophtheirus salmonis *in Atlantic salmon

**DOI:** 10.1186/1471-2156-10-20

**Published:** 2009-04-27

**Authors:** Karim Gharbi, Kevin A Glover, Louise C Stone, Elizabeth S MacDonald, Louise Matthews, Unni Grimholt, Michael J Stear

**Affiliations:** 1 Institute of Comparative Medicine, University of Glasgow, Glasgow, G61 1QH, UK; 2 Institute of Marine Research, N-5817 Bergen, Norway; 3 CEES, Department of Biology, University of Oslo, Oslo, Norway; 4 School of Biosciences, University of Birmingham, Birmingham, B15 2TT, UK

## Abstract

**Background:**

Genetic variation has been shown to play a significant role in determining susceptibility to the salmon louse, *Lepeophtheirus salmonis*. However, the mechanisms involved in differential response to infection remain poorly understood. Recent findings in Atlantic salmon (*Salmo salar*) have provided evidence for a potential link between marker variation at the major histocompatibility complex (MHC) and differences in lice abundance among infected siblings, suggesting that MHC genes can modulate susceptibility to the parasite. In this study, we used quantitative trait locus (QTL) analysis to test the effect of genomic regions linked to MHC class I and II on linkage groups (LG) 15 and 6, respectively.

**Results:**

Significant QTL effects were detected on both LG 6 and LG 15 in sire-based analysis but the QTL regions remained unresolved due to a lack of recombination between markers. In dam-based analysis, a significant QTL was identified on LG 6, which accounted for 12.9% of within-family variance in lice abundance. However, the QTL was located at the opposite end of *DAA*, with no significant overlap with the MHC class II region. Interestingly, QTL modelling also revealed evidence of sex-linked differences in lice abundance, indicating that males and females may have different susceptibility to infection.

**Conclusion:**

Overall, QTL analysis provided relatively weak support for a proximal effect of classical MHC regions on lice abundance, which can partly be explained by linkage to other genes controlling susceptibility to *L. salmonis *on the same chromosome.

## Background

The salmon louse, *Lepeophtheirus salmonis*, is a parasitic copepod (family Caligidae) that feeds on the mucous, tissue, and blood of salmonid fish [1-3]. Infected hosts can suffer from mild skin damage to stress-induced death, including reduced growth, osmoregulatory stress, and secondary infections [1]. In Northern Europe, *L. salmonis *has become endemic on farmed Atlantic salmon (*Salmo salar*), with disease outbreaks initially resulting in heavy losses [1,4]. Pest management programs now implemented across most of the industry have significantly reduced mortality due to infestations but farmed populations are still exposed to high infection pressure and require frequent delousing treatment [4].

Atlantic salmon is generally regarded as more susceptible to *L. salmonis *than other salmonid hosts [5,6]. However, some degree of heritable variation has also been reported in levels of infestation among individuals [7,8]. For example, Glover *et al*. [7] observed a heritability of 0.07 for lice abundance in a single fish cohort naturally exposed to *L. salmonis*. In a separate study, Kolstad *et al*. [8] estimated average heritabilities of 0.14 and 0.26 for susceptibility to infection in multiple year-classes following natural and experimental challenges, respectively. Together, these results confirm earlier evidence of genetic variation for susceptibility to *L. salmonis *in Atlantic salmon [9] and indicate that host genes play a significant role in determining infection levels.

The underlying mechanisms involved in differential susceptibility to initial infection and subsequent interactions with *L. salmonis *remain poorly understood. Species differences in host response have been linked to epidermal inflammation [5,6,10] but the functional basis of differences in the susceptibility of individual hosts have yet to be elucidated. A possible link has recently been identified between marker variation at major histocompatibility complex (MHC) genes and lice abundance in families of Atlantic salmon naturally infected with *L. salmonis *[11]. Significant associations were found with both classical MHC class I (*UBA*) and II (*DAA*) in multiple families, indicating that susceptibility to infection may be modulated by genes within the MHC. Alternatively, the MHC may merely act as a marker of host susceptibility through linkage to other genes controlling lice abundance on the same chromosome.

In this study, we used quantitative trait locus (QTL) analysis to elucidate the relationship between MHC variation and susceptibility to *L. salmonis*. We reasoned that, if MHC genes have sizeable effects on susceptibility to infestation, QTL analysis of lice abundance in infected siblings should identify the MHC regions as contributing to a significant proportion of family variance. A confounding factor with this approach, however, is the strong suppression of male recombination in Atlantic salmon, which can cause chromosomally distant regions to co-segregate from sires to offspring [12,13]. Thus, we expected that sire-based analysis would broadly confirm the presence of QTL influencing lice abundance on the MHC chromosomes, while dam-based analysis would allow us to better resolve the location of QTL effects.

## Results

### Overview of QTL families

Body weight and lice abundance in the families selected for QTL analysis are summarized in Table 1. Further information on lice abundance in each family is provided in Additional file 1 . Each family was chosen based on previous results [11] suggesting significant associations between lice abundance and marker variation at *UBA *(family 4), *DAA *(family 6) or both (family 3). Overall, lice abundance ranged from 4 to 38 parasites per fish (mean = 19.1 ± 5.5), with no significant differences between families (two-sample t-test, p > 0.05). As noted previously [11], lice abundance was positively correlated with body weight in all three families (Pearson's correlation test, p < 0.05).

**Table 1:** Overview of QTL families

**Family**	**N**	**Body weight (g)**		**Lice abundance (fish^-1^)**		**Corr.**
		range	mean (sd)	range	mean (sd)	
Family 3	73	142–1010	513.0 (162.8)	4–31	19.6 (5.4)	0.42**
Family 4	109	112–874	445.7 (148.6)	7–38	19.4 (5.8)	0.20*
Family 6	112	228–654	447.4 (91.6)	7–32	18.6 (5.4)	0.20*

**Notes**. Corr.: Pearson's correlation coefficient between body weight and lice abundance (* p < 0.05, ** p < 0.001); distribution plots of lice abundance in each family are provided in Additional file 1.

### Sex-specific maps of MHC chromosomes

MHC class I and II are unlinked in Atlantic salmon, with each region present in duplicate copies on different chromosomes [14-16]. The major class I locus (*UBA*) has been shown to reside on a small chromosome [14,15], which in the current map is represented by LG 15 [17]. Classical MHC class II genes, including *DAA*, have been linked to markers currently assigned to LG 6 [14,16,18].

**Figure 1:**
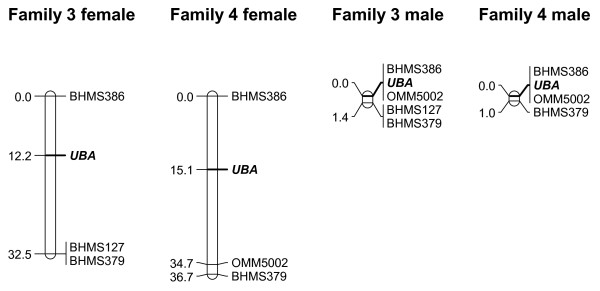
**Sex-specific maps of LG 15 in families 3 and 4**. Linkage maps are shown for each sire (right) and dam (left). Absolute map distances are given in cM. The position of *UBA *is highlighted in bold.

**Figure 2:**
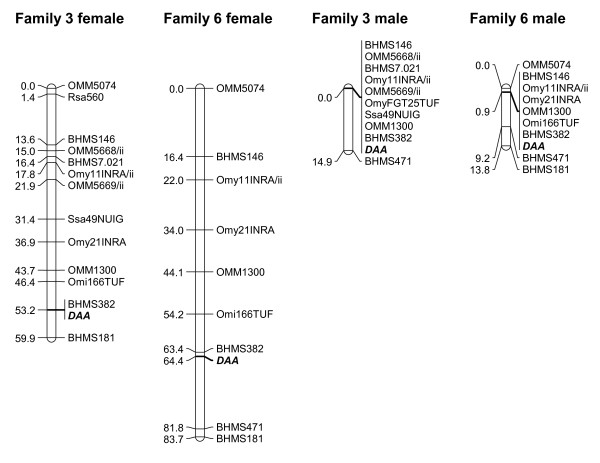
**Sex-specific maps of LG 6 in families 3 and 6**. Linkage maps are shown for each sire (right) and dam (left). Absolute map distances are given in cM. The position of *DAA *is highlighted in bold.

Dam-based maps of LG 15 in families 3 and 4 were relatively short, with *UBA *in the central region (Figure 1). In sires, map distances were considerably reduced due to strong recombination suppression, resulting in a highly biased female-to-male recombination ratio across the linkage group (28.8:1). Linkage analysis of LG 6 markers in families 3 and 6 resulted in generally longer maps, suggestive of a larger chromosome (Figure 2). In dams, *DAA *mapped towards the distal end of the chromosome, near marker BHMS382. In sires, recombination was again strongly reduced across the most part of the linkage group. However, recombination rates in the distal region were comparable between sexes, resulting in a relatively moderate female-to-male recombination ratio overall (5.0:1).

### QTL scan of MHC chromosomes

**Figure 3:**
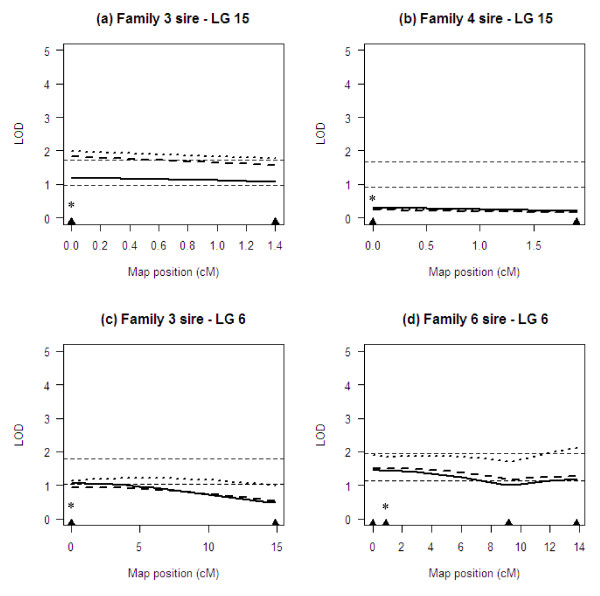
**Sire-based QTL scans of LG 6 and LG 15 for lice abundance**. Multipoint LOD score profiles were generated using alternative covariate parameters for the effect of putative sex on lice abundance (solid line: no effects; dashed line: additive effects; dotted line: interactive effects). Triangles along the x axis indicate marker positions. Asterisks show the location of *UBA *and *DAA *on LG 15 and 6, respectively. Horizontal lines across each plot indicate LOD significance thresholds (lower threshold: chromosome-wide significance; upper threshold: experiment-wide significance).

**Figure 4:**
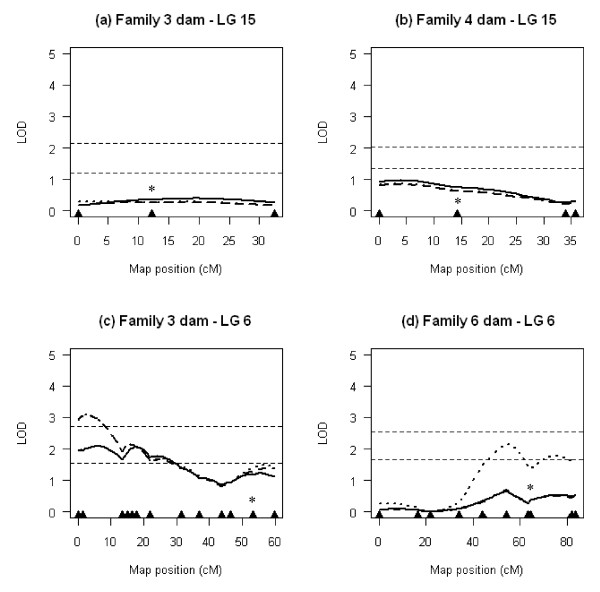
**Dam-based QTL scans of LG 6 and LG 15 for lice abundance**. Multipoint LOD score profiles were generated using alternative covariate parameters for the effect of putative sex on lice abundance (solid line: no effects; dashed line: additive effects; dotted line: interactive effects). Triangles along the x axis indicate marker positions. Asterisks show the location of *UBA *and *DAA *on LG 15 and 6, respectively. Horizontal lines across each plot indicate LOD significance thresholds (lower line: chromosome-wide significance; upper line: experiment-wide significance).

Single-QTL scans for lice abundance based on sex-specific maps of LG 15 and LG 6 are shown in Figures 3 and 4, respectively. Three different profiles are shown for each parent, with each analysis corresponding to an alternative covariate model for the effect of putative sex on lice abundance (see Methods). Due to the lack of recombination between markers, QTL profiles derived from sire-based analysis were relatively flat, with little variation across map intervals (Figure 3). Nevertheless, LOD values exceeded the 95% chromosome-wide significance level on LG 15 in the sire of family 3 and LG 6 in the sire of family 6, irrespective of the covariate model (Figures 3a and 3d). In addition, markers on LG 6 reached chromosome-wide significance in the sire of family 3 when sex was included as an interactive covariate (Figure 3c). No significant QTL effects were found on LG 15 in the sire of family 4 (Figure 3b). At the experiment-wide threshold, QTL effects on LG 15 in the sire of family 3 and LG 6 in the sire of family 6 remained significant when sex was used as an interactive covariate (Figure 3a and 3d). However, it was not possible to assign either QTL to a specific chromosome region as confidence intervals spanned the entire length of the linkage group.

In dam-based analysis, QTL effects above the chromosome-wide significance threshold were detected on LG 6, but not on LG 15 (Figure 4). In the dam of family 6, two QTL peaks were detected on either side of the MHC class II region in the QTL-by-sex interaction model (Figure 4d). However, neither peak reached experiment-wide significance. In the dam of family 3, all three models were significant at the chromosome-wide threshold, although the QTL peak was higher and better resolved when sex was fitted as a covariate (Figure 4c). The highest peak (LOD = 3.07) remained significant at the experiment-wide threshold, with a predicted location at position 3.0 cM, near marker Rsa560. Bayesian credible intervals estimated with a posterior probability of 95% included the proximal region of LG 6 from positions 0.0 to 28.0 cM.

### QTL modelling of lice abundance in family 3

To further characterize QTL effects identified on LG 6 in the dam of family 3 (Figure 4b), we modelled lice abundance as a function of QTL genotypes, body weight, and sex (Table 2). Overall, the fitted model explained 38.4% of the total family variance in lice abundance, with QTL genotypes accounting for an estimated 12.9% of the total variation. Associated QTL effects were approximately 4 lice or 0.75 standard deviations. Sex and body weight accounted for a further 10.6% and 9.6% of family variance, with estimated effects of 3.6 lice and 1.0 louse per 100 g, respectively.

**Table 2:** Dam-based QTL modelling of lice abundance in family 3

	**df**	**SS**	**LOD**	**effects (sd)**	**% var**	**p-value**
***Full model***						
**Model**	3	817.8	7.8		38.4	1.9 e-07
**Error**	70	1312.5				
***Drop-one-term analysis***						
**c6loc3d**	1	275.9	3.1	4.07 (1.06)	12.9	2.7 e-04
**Sex**	1	225.5	2.5	3.61 (1.04)	10.6	9.0 e-04
**Body weight**	1	205.1	2.3	0.01 (0.03)	9.6	1.5 e-03

**Notes**. Degrees of freedom (df), sum of squares (SS), LOD scores (LOD), estimated effects (sd: standard deviation), percent of variance explained (% var), and p-values in drop-one-term analysis were obtained by comparing the full model to the reduced model with the term in the first column dropped; c6loc3d denotes predicted QTL genotypes at position 3.0 cM of LG 6 in dam-based analysis.

## Discussion

### Association of MHC genotypes with lice abundance

Significant QTL associations between MHC genotypes and lice abundance were confirmed in families 3 and 6, but not in family 4. In both cases, QTL associations were detected in sires but not in dams, although we did find suggestive evidence of a possible QTL near the MHC class II region on LG 6 in the dam of family 6. However, this QTL was only significant at the chromosome-wide level and should be regarded as tentative until additional dams are examined. Conversely, the QTL detected on LG 6 in the dam of family 3 was significant at the experiment-wide threshold but a causal link with MHC variation is unlikely due to its predicted location at the opposite end of MHC class II (see below). Overall, therefore, we were unable to corroborate that either MHC region has a significant effect on lice abundance, despite sire-based analysis pointing to potential QTL variation on both MHC chromosomes.

### Detection of a QTL for lice abundance on LG 6

The most important finding of this study was the detection of a QTL influencing lice abundance within the proximal region of LG 6. The QTL peak was located at position 3.0 cM, with a 95% posterior probability that the QTL region lies within 28 cM of the most proximal marker (OMM5074). The MHC class II region, on the other hand, was located towards the opposite end of the linkage group at position 53.2 cM. Taken together, these results strongly suggest that the observed QTL variation is unlikely to be caused by *DAA *or other genes within the MHC class II complex, although we cannot exclude the possibility that the QTL region contains regulatory elements that can modulate MHC class II activity, for example through transcriptional control.

Accounting for the effect of body weight and sex, the QTL on LG 6 explained nearly 13% of the total variance in lice abundance among progeny in family 3. This level of explained variation is generally regarded as moderate in QTL studies, although in this case it appears to represent a relatively large proportion of the observed heritability for lice abundance (0.06 to 0.19) in naturally infected fish [7,8]. If this estimate was representative of the breeding population, the QTL would account for much of the genetic variation in lice abundance, at least in some families. However, gene frequencies at the QTL need to be taken into account and our results are also consistent with a relatively rare QTL allele accounting for a smaller proportion of the total genetic variance across the population.

Linkage groups 6 and 15 were specifically targeted in this study because they carry the classical MHC class I and II genes previously linked to differences in susceptibility to *L. salmonis *[11]. Other linkage groups were not examined, with the exception of one marker on the sex chromosomes (LG 1). However, it is possible that important QTL influencing lice abundance also exist elsewhere in the salmon genome. Further QTL studies should therefore focus on fine-mapping the QTL identified on LG 6 as well as searching for additional QTL on other linkage groups.

### Effect of sex on lice abundance

Sex was not directly recorded in the fish used in this study but it was possible to differentiate between males and females within each family using a sex-linked marker (see Methods). However, since linkage phases were unknown, it was not possible to assign male or female status to progeny inheriting alternative sire alleles at the sex marker (i.e., progeny were assigned to groups of opposite sex but actual sex was unknown). Nevertheless, sex predicted with this method accounted for over 10% of the variation in lice abundance in family 3, indicating that males and females may have different susceptibility to *L. salmonis*.

To our knowledge, only a few studies have explicitly examined the effect of sex or maturation on lice abundance [19,20]. No differences were observed between males and females, although fish size, which is known to vary between sex, has been found to be an important factor in determining lice abundance [7]. In this study, body weight was generally higher in the gender with higher lice numbers (537.2/481.9 g versus 21.0/17.3 lice, respectively) but size alone cannot fully account for these differences as lice abundance was corrected for body weight in QTL analysis (see Methods). Sex differences in parasite infections are not uncommon in vertebrates and may be mediated by a variety of ecological (e.g., behavioural) and physiological (e.g., hormonal) causes [21]. Whether such factors may be involved in susceptibility to *L. salmonis *is currently unknown and will require a more detailed comparison of host response in males and females.

Sex differences among siblings may also be caused by a sex-linked QTL, rather than sex itself. In this context, it is interesting to note that the sex linkage group (LG 1) and LG 6 share extensive homologous regions inherited from a whole-genome duplication in the salmonid ancestry [12]. Thus, it is possible that duplicated QTL regions with ancestrally conserved effects on susceptibility to parasite infection may have been retained on both linkage groups through evolutionary time. Evidence for the apparent retention of QTL function between duplicated chromosomes has previously been found for life-history traits [22-24] but this would be the first example of a duplicate QTL region for a disease trait.

### Functional basis of differential susceptibility to *L. salmonis*

It is now apparent from the present and previous studies [7,8,11] that host genes are important determinants of susceptibility to *L. salmonis *in Atlantic salmon. However, further research is required to elucidate the functional basis of differential susceptibility to infection among individuals. Possible mechanisms include a variety of host factors, such as non-specific defences [25], adaptive immunity [26], mucous biochemistry [6], and skin thickness [1]. Perhaps most significant are the inflammatory differences associated with variation in susceptibility to initial infection among salmonid species [5,6,10]. Atlantic salmon, for example, exhibit only minor tissue response to the presence of *L. salmonis*, regardless of developmental stage [5]. By contrast, *L. salmonis *larvae attached to coho salmon (*Oncorhynchus kisutch*) or pink salmon (*Oncorhynchus gorbuscha*) elicit a well-developed inflammatory reaction and hyperplesia of the epithelium around the site of attachment [5,10]. In addition, enhanced resistance to *L. salmonis *in pink salmon has been linked to earlier onset and greater up-regulation of pro-inflammatory genes, such as interleukin-1β (IL-1β) and tumour necrosis factor-α (TNF-α) [10]. In Atlantic salmon, *L. salmonis *also induces changes in the expression of pro-inflammatory and other immune-related genes [27-29] but it is currently unknown whether transcriptional regulation can mediate differential susceptibility to infection.

## Conclusion

Overall, QTL analysis provided relatively weak support for a proximal effect of classical MHC regions on lice abundance in the families examined in this study. The identification of a QTL for lice abundance at the opposite end of MHC class II on LG 6 in dam-based analysis indicates that the reported association with MHC variation may, at least in part, reflect linkage disequilibrium. However, sire-based analysis remained inconclusive and the existence of MHC QTLs segregating in the sire line cannot be excluded using this approach. Nevertheless, the discovery of a QTL influencing susceptibility to *L. salmonis *represents a significant step toward a better understanding of the host response to infection, which in turn will assist in developing improved methods of parasite control in salmon farming.

## Methods

### Fish samples

The samples used in this study were part of an infection trial conducted on the west coast of Norway in 2004 [11]. In brief, 15 full-sib families (age 1+) derived from the Aqua Gen AS breeding programme were transferred to seawater in May 2004 and held in a single net-pen without delousing treatment for a period of 6 months. In October 2004, fish were sampled by groups of 5–10 individuals into large buckets containing an overdose of anaesthetic. Sedated fish were killed by a sharp blow to the head, placed into individual buckets, and immediately transferred to the laboratory for examination. Weight and lice abundance were recorded for a total of 1,342 individuals. A random sample of approximately 1,000 fish from 11 families were subsequently genotyped at simple sequence repeat (SSR) markers located within the 3'-UTR of the MHC class I (*UBA*) and II (*DAA*) alpha chain genes. A subset of 294 fish from three families were selected for QTL analysis in this study based on initial results indicating significant associations between lice abundance and MHC variation (see Table 1).

### Parasite data

Parasite data consisted of whole-body lice counts recorded from each individual at the time of sampling. Both *L. salmonis *and *Caligus elongatus *were present on the infected fish but *C. elongatus *was excluded from analysis due to low prevalence [11]. In addition, developmental stage was not recorded as initial inspection of infected samples indicated that most parasites were adults and pre-adults [11]. Thus, in the context of this study, lice abundance represents the total number of mobile *L. salmonis *recovered per fish.

### Marker genotyping

Genomic DNA was isolated in 96 well-plate format using QIAGEN DNAeasy kit. SSR markers located on LG 6 and LG 15 were selected based on data obtained from the Salmon Genome Project [30], the cGRASP database [17], and recent genotyping in the SALMAP families [31]. Between 4 and 19 markers were genotyped in each family. Polymerase chain reactions (PCR) were carried out in 15-μl volumes containing 1× GoTaq reaction buffer (Promega), 1.5 mM MgCl_2 _(Promega), 300 nM forward primer end-labelled with HEX, FAM, or TAMRA (MWG Biotech), 300 nM reverse primer (MWG Biotech), 200 μM dNTPs (Invitrogen), 0.25 units GoTaq polymerase (Promega), and 30 ng genomic DNA. Cycle amplifications were performed in PTC-200 or Dyad thermocyclers (MJ Research) for 5 min at 96°C; 5 cycles of 1 min at 96°C, 30 sec at 48–62°C, 30 sec at 72°C; 30 cycles of 30 sec at 95°C, 30 sec at 53–58°C, 30 sec at 72°C; 5 min at 72°C; and a final soak at 10°C (see Additional file 2 for details of primer sequences and optimized PCR conditions). PCR products from groups of up to three markers with different dye labels were diluted 1:2–1:4 into distilled water and 2 μl of each diluted sample were added to 0.75 μl GeneScan 400HD ROX size standard (Applied Biosystems) and HiDi formamide (Applied Biosystems) up to a 20-μl final volume. Samples were denatured at 95°C for 5 min prior to genotyping on an ABI PRISM 3130 *xl *Genetic Analyzer (Applied Biosystems). Genotyping files were analyzed using GeneMarker version 1.70 (SoftGenetics).

### Linkage analysis

Linkage analysis was performed with CARTHAGENE version 1.0R [32] using genotype data converted to a backcross format. As grandparent genotypes were unknown, linkage phases were determined retrospectively by examining assortment of alleles among linked markers. Graphic representations of linkage groups were generated with MAPCHART version 2.1 [33] using raw recombination fractions as estimates of map distances.

### QTL analysis

Parasite data were checked for deviation from normality prior to QTL analysis using the Shapiro-Wilk test (p = 0.05). As a result, one individual identified as an outlier was excluded from family 6. All QTL analyses were carried out using simple interval mapping (SIM) in R/qtl version 1.09 [34]. Single-QTL models were fitted in each parent using the Haley-Knott regression method [35] with conditional genotype probabilities calculated at 1 cM intervals and a constant genotyping error rate of 1%. Body weight was used as a covariate in all QTL models to control for the effect of fish size on lice abundance [11]. In addition, sex was included as an optional covariate in alternative QTL models using genotypes from a duplicated SSR marker (Omy11INRA/i) closely linked to the major sex-determining locus on LG 1 [36]. Three separate QTL models were produced for each parent by changing covariate parameters for sex (i.e., null, additive, and interactive, respectively).

Chromosome-wide thresholds for QTL significance (p-value = 0.05) were estimated empirically by permutation sampling with 1,000 replicates. Experiment-wide significance thresholds were derived from permutation estimates by dividing the nominal p-value by the total number of chromosomes examined in the study (i.e., 0.05/8~0.006). Confidence intervals for the location of significant QTL were estimated using Bayesian credible intervals with a 95% probability coverage (see R/qtl documentation).

Further QTL modelling was carried out by analysis of variance (ANOVA) using the *fitqtl *function in R/qtl. Putative QTL genotypes were added to the model using conditional genotype probabilities calculated at 1 cM intervals and a genotyping error rate of 1%. Estimates of QTL effects and percentage of variance explained were obtained by comparing the full model to the sub-model without QTL. A similar approach was used for estimating covariate effects.

## Authors' contributions

KG conceived the QTL study, supervised marker genotyping, conducted data analysis, and drafted the manuscript. KAG collected infection data and conducted the MHC study. LS and EM carried out marker genotyping and initial data analysis. UG contributed in MHC analysis. MS and LM assisted in data analysis and drafting the manuscript. All authors read and approved the final manuscript.

## Supplementary Material

Additional file 1 **Distribution of lice abundance in QTL mapping families**. Density plots of the distribution of lice abundance in each family and in the pooled sample. Click here for file

Additional file 2 **Marker information**. GenBank accessions, primer sequences, and optimized PCR conditions for each SSR marker. Click here for file
