# Supplementary figures and images for: Genetic dissection of MHC-associated susceptibility to Lepeophtheirus salmonis in Atlantic salmon
Source: BMC Genet. 2009 Apr 27;10:20. doi: 10.1186/1471-2156-10-20 (PMC2680909; doi:10.1186/1471-2156-10-20)

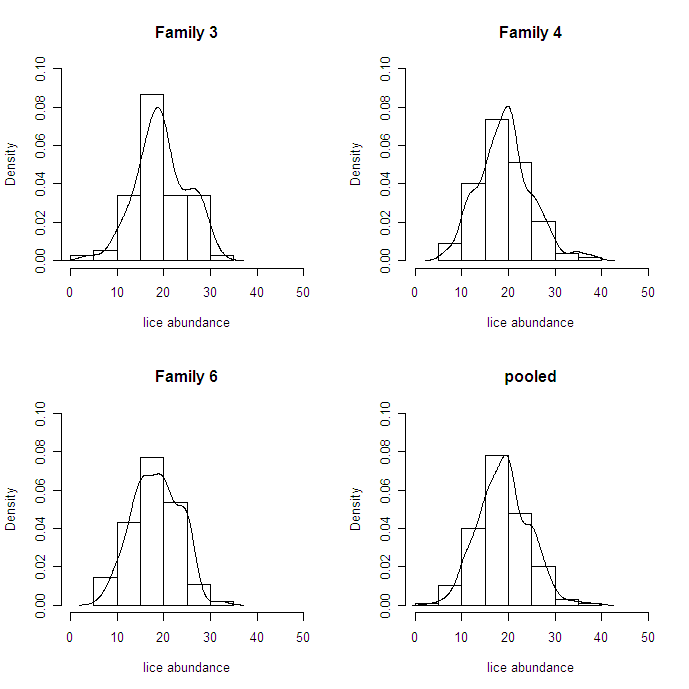

Supplement: Additional file 1 — Distribution of lice abundance in QTL mapping families. Density plots of the distribution of lice abundance in each family and in the pooled sample. [file 1471-2156-10-20-S2.doc]
