# Supplementary material for: Genetic dissection of MHC-associated susceptibility to Lepeophtheirus salmonis in Atlantic salmon
Source: BMC Genet. 2009 Apr 27;10:20. doi: 10.1186/1471-2156-10-20 (PMC2680909; doi:10.1186/1471-2156-10-20)
Supplement: Additional file 2 — Marker information. GenBank accessions, primer sequences, and optimized PCR conditions for each SSR marker. [file 1471-2156-10-20-S1.doc]

## Additional Table 1 - SSR marker information

| **Marker name** | **Accession no.** | **LG^1^** | **Primer sequences (5’ → 3’)** | **Label** | **Ta^2^** | **Reference** |
| --- | --- | --- | --- | --- | --- | --- |
| BHMS382 | AF256710 | AS-06 | F-TTG TGG AGT ATT TAG CAA TC  R-ATT GGC AGA CAT GCA CTC | HEX | 54 | [1] |
| BHMS471 | AF256723 | AS-06 | F-TCT GTC CGT CCT GCA TAC  R-CTT ATG TTG TGT TGT GCT G | HEX | 48 | [1] |
| CL4778 | - | AS-06 | F-GAG GAT ACT GCC ACT TCA ACA  R- ACG GTC CCA CCT TAC CAT AAA | HEX | 58 | [2] |
| OMM5669 | BX301679 | AS-06^3^ | TCTCTGAGCAAGGACCCTGC  GCCCGACAATAGGTTAAGGC | HEX | 50 | - |
| OMM1300 | AF470056 | AS-06 | F-GAG TCA CAC GAT AAT ACC ACA GCG ATA  R-ATA TGC AGC AGA AGG TGG AAA CTT GA | HEX | 54 | [3] |
| Ssa49NUIG | AF420528 | AS-06 | AGT GCC GAG GCG GTG GCA  CGG TGA GGT TGG ACC GTG G | HEX | 56 | - |
| BHMS7.021 | AF256837 | AS-06 | F-AGC ACT GAG ACT TCT ATC AG  R-AGA GAT CCA GGT AAA CAG AC | FAM | 54 | [1] |
| BHMS146 | AF256666 | AS-06 | F-GTC ATG CAA ACA AAC ATT GG  R-ACA AAT CCC CCG CAC ACA C | FAM | 58 | [1] |
| BHMS181 | AF256674 | AS-06 | F-AAA GAC ACG GAG CAA GGC  R-AAG ACA GGA GTC TGG GTG | FAM | 56 | [1] |
| OMM5074 | CA348721 | AS-06 | F-TCG CTT TGG GTA GAA GTT GCC TTT AAC  R-AAC ATT AAG AAC GAG TGG AAT CAC GC | FAM | 56 | [4] |
| Omy11INRA | - | AS-06^3^ | F-GTT CAA GTC AAA GCA GCT GG  R-CCA GGG CAT GGC TTT GG | FAM | 62 | [5] |
| Rsa560 | AY544094 | AS-06 | F-TGA GTA GAT GTT CTG AGA CG  R-TGG TAA GAG AGA GTG TTA GTG | FAM | 56 | [1] |
| Omi166TUF | AB213246 | AS-06 | F-AAG TCC TTC AAG TCT GTC TCC G  R-TGC TTA CAA AGG AGC AAA TGG | TAMRA | 54 | T. Hara & N. Okamato |
| OMM5668 | BX311884 | AS-06^3^ | F-TGGACAACTTCAGCAAGGACC  R-TGGTGACAGTTTCTGCTGAACC | TAMRA | 54 | - |
| OmyFGT25TUF | - | AS-06 | F-GTC GCA GTC TGA ACT CAC CA  R-CAG GTG GTT GTG TGT CCT TG | TAMRA | 52 | T. Sakamoto |
| Omy21INRA | - | AS-06 | F-GCA TTG GCG TAA TGA GAA GG  R-CTG ACG GAC ATA TCA GCC C | TAMRA | 50 | [5] |
| UBA | EF210363 | AS-15 | F-CCA CTG GCA GAC TGA ATC TGG  R-AGA CAC TCA GGG AGG CTG AGG | HEX | 60 | this study |
| BHMS127 | AF256662 | AS-15 | F-TTA CAC AGC CCT GCT CAC  R-TCC TGT CAC ACT CAC TAC C | HEX | 54 | [1] |
| BHM386 | AF256712 | AS-15 | F-CGT TAA AAC CCC GTG GAG  R-GAC TAA AAA GCG TCT GGC | FAM | 54 | [1] |
| OMM5002 | CO805108 | AS-15 | F-GGG CTT CCT GGA GGA CTA CTT TA  R-GCC CTG ACA GAC AGC AAC ATA TAG | TAMRA | 58 | [4] |
| BHMS379 | AF256708 | AS-15 | F-GAA CAA CTT CAG AAC TTG AC  R-CGC CTC ATA GCT GAT ATT TAA C | TAMRA | 54 | [1] |

**Table legend**. ^1^ Linkage group assignment based on data from cGRASP [6] and the Salmon Genome Project [1] ^2^ Annealing temperature for PCR amplification ^3^ Markers that amplify duplicated loci in the salmon genome.
